# Cannabis and Cannabidiol, GLP-1 Receptors, and Autophagy: The Burgeoning Link Between Cognitive Neurodegeneration With Alzheimer’s Disease and Metabolic Disorders

**Published:** 2026-01-20

**Authors:** Kenneth Maiese

**Affiliations:** 1 Cellular and Molecular Signaling, New York, NY 10022, USA

## The Link Between Cognitive Loss and Metabolic Dysfunction

According to the World Health Organization, diseases of the nervous system now impact over 3.4 billion individuals and represent more than forty-two percent of the global population [1]. Neurological conditions lead to greater than 7 million deaths on an annual basis and result in 435 million disability-adjusted life years. As a result, disorders of the nervous system are the leading causing of disability throughout the globe [2,3]. The top neurological disorders leading to illness are stroke, neonatal encephalopathy, migraine, cognitive loss that includes Alzheimer’s disease (AD), diabetic neuropathy, meningitis, epilepsy, preterm birth neurological complications, autism spectrum disorders, and tumors of the nervous system. As of the year 2019, the cost for treating and caring for neurological disorders reached $1.7 trillion United States dollars (USD) and has been growing 3.5% annually since the year 2000. Interestingly, cognitive disorders associated with dementia and stroke can account for the largest costs [1].

In regard to memory and cognitive loss, disorders involving dementia are presently the seventh cause for death and it is expected that over the next 25 years more than 160 million individuals worldwide will have dementia that includes diseases such as AD [4,5]. In individuals with dementia, at least 60 percent suffer from the sporadic form of AD and 10 percent have an age greater than 65. With at least 7 million individuals in the United States currently diagnosed with AD, the prevalence of the disorder is expected to increase to 30 million individuals over the next 20 years. The expected increase in AD corresponds with the observed increase in lifespan throughout the world such that it is predicted most individuals will reach the age of 80, a contrast over the prior 50 years that predicted lifespan has doubled for those individuals reaching 65 years of age. Yet, the increases observed in lifespan also account for processes tied to degeneration and aging. Destabilization of cellular telomeres (TLs) results in cellular senescence and cellular degeneration. TLs are formed from deoxyribonucleic acid (DNA) present on chromosome ends and maintain DNA integrity, cellular reproduction, and cellular survival. With aging, TL dysfunction ultimately leads to cellular senescence which results in organ repair failure, production of reactive oxygen species and oxidative stress, mitochondrial dysfunction, and cellular metabolic disorders.

Metabolic disorders, that include diabetes mellitus (DM), are intimately tied to the onset of neurodegenerative disease and cognitive loss. Similar to neurodegenerative disorders and cognitive impairment, metabolic disease and DM are increasing in prevalence globally with the number of individuals with DM at 420 million and expected to reach 700 million individuals in the year 2045. More than 50 percent of the 4 million deaths that occur per year with DM affect individuals younger than seventy years of age. DM is a chronic and multi-systemic disorder that leads to cardiac disease, retinal cell loss, renal impairment, liver compromise, cerebral ischemia, and dementia. Financial costs for the care of individuals with DM are significant reaching $760 billion USD with an additional $70 billion USD necessary for individuals with disability and functional loss. Just in the US, these costs are more than 17 percent of the Gross Domestic Product for DM care. Multiple factors contribute to metabolic disease progression that include hypertension, high serum cholesterol, tobacco and alcohol consumption, limited physical activity, and obesity. Increased body weight with obesity affects insulin sensitivity, glucose intolerance, cerebral blood flow, aging progression, and susceptibility to infection, such as with coronavirus disease 2019 (COVID-19) in DM patients.

At the cellular level, metabolic dysfunction and DM promote oxidative stress, loss of stem cell function, inflammation, glymphatic pathway dysfunction, absence of mitochondrial homeostasis, endothelial cell impairment, neuronal cell injury, myelin degradation, and changes in neurotransmitter release [6–9]. As a result of these cellular processes, cognitive impairment and dementia can ensue with DM resulting in memory impairment and AD. Late-onset AD also is associated with the metabolic pathway of the *ε*4 allele of the apolipoprotein E (*APOE-ε4*) gene with the risk for developing AD becoming more than 20 times greater in individuals harboring two APOE-*ε*4 alleles [5,10–12]. APOE is critical for metabolic cellular function and is produced in the liver to control the homeostasis of lipids through the transport of cholesterol, triglycerides, and phospholipids. Although some subtypes of APOE can remove potentially toxic *β*-amyloid (A*β*) in the brain during AD through apoptotic programmed cell death and the exposure of cellular phosphatidylserine (PS) membranes that can attract microglia for toxin removal, APOE-*ε*4 does not inhibit A*β* aggregation and may add additional disability by permitting viral antigen infection. This can lead to cerebral microhemorrhages during severe acute respiratory syndrome (SARS-CoV-2) exposure with coronavirus disease 2019 (COVID-19).

## Targeting Dementia and Metabolic Disease With Cannabis and Cannabidiol

Both cognitive neurodegenerative disorders, such as AD, and metabolic disease that includes DM are chronic progressive disorders that call for innovative therapies to address the intimate link between these disease entities. Currently, therapies for dementia and AD can help reduce memory impairment progression, such as with the application of cholinesterase inhibitors [13,14]. In addition, recently U.S. Food and Drug Administration (FDA) approved immunotherapy treatments that reduce brain A*β* deposition as well as proposed therapies that limit tau accumulation can slow memory decline in some patients, but do not halt disease progression and may lead to brain microhemorrhages [2,15–17]. Similarly, trusted methods to treat DM involve hypoglycemic pharmaceutical treatments, maintaining serum glucose homeostasis, nutritional guidance, exercise, and weight reduction, but metabolic disease can continue with progressive organ dysfunction [18]. Going forward, addressing the interplay between neurodegenerative disease and metabolic disorders may provide innovative avenues to treat the underlying processes of these chronic disease entities.

In this regard, cannabidiol (CBD) may represent a novel agent for the treatment of both cognitive loss and metabolic disease. CBD is one of over 100 cannabinoid compounds in Cannabis, a genus of flowering plants in the Cannabaceae family [19]. CBD is structurally similar to Cannabis tetrahydrocannabinol (THC) and is a nonpsychotropic phytocannabinoid and a multi-ring phenolic compound. Although at increased temperatures, CBD may control the efficacy of THC and psychotropic effects, under non-extreme conditions CBD and THC do not hold the same psychological activity such that CBD is nonpsychotropic and favored for patient treatment regimens. CBD can be administered by multiple routes that include oral sublingual tissue absorption, vapor inhalation, topical application, and oral intake. At present, CBD is approved by the FDA for the treatment of pediatric epilepsy in Lennox-Gastaut syndrome and Dravet syndrome [20].

## Active Considerations of Cannabidiol for Alzheimer’s Disease and Diabetes Mellitus

**Fig. 1.**
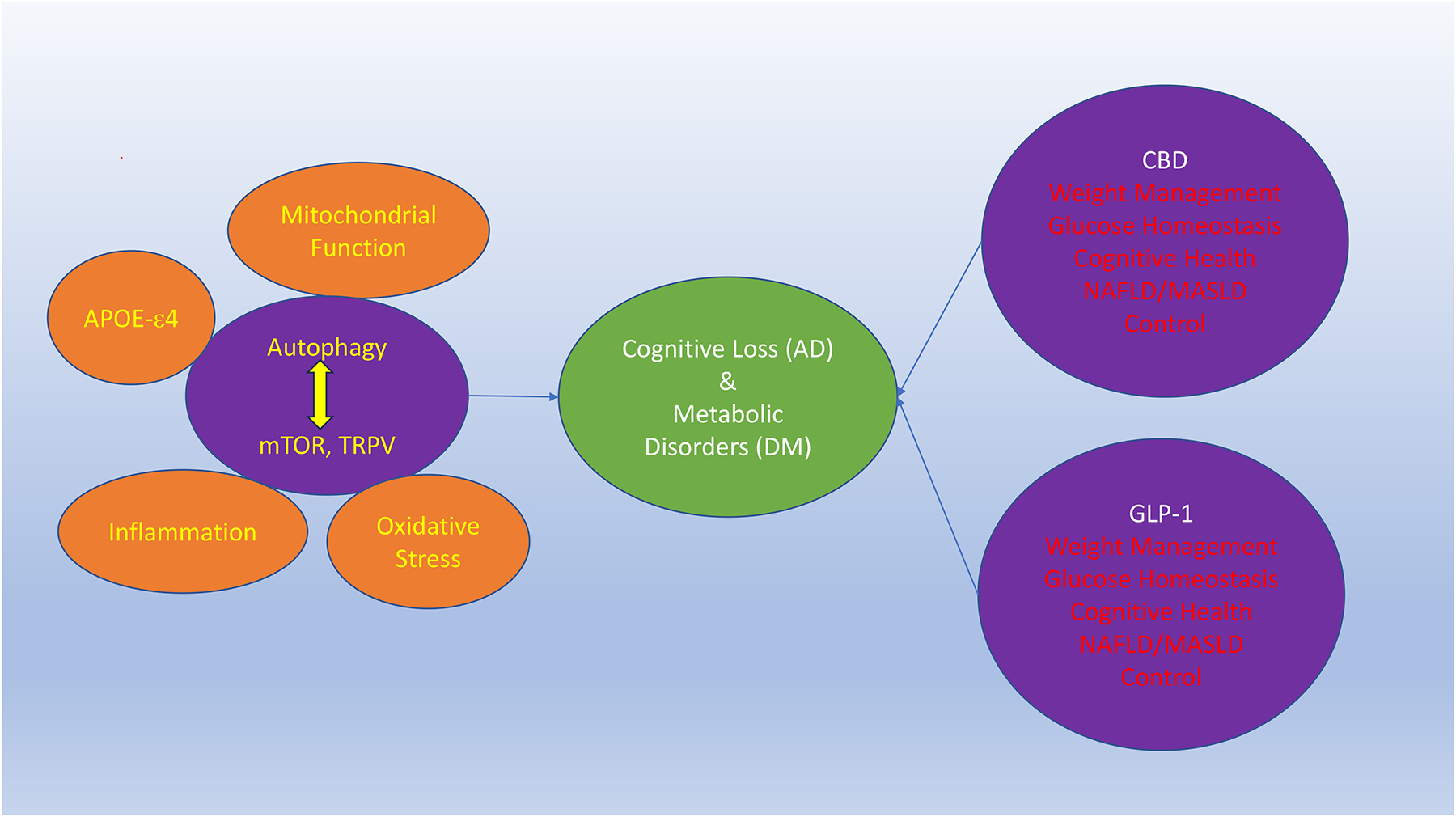
Cannabidiol and GPL-1 receptor agonists share common cellular mechanisms to address neurodegenerative cognitive loss and metabolic disease. Cannabidiol (CBD) and glucagon-like peptide-1 (GLP-1) receptor agonists are exciting treatments to address both neurodegenerative cognitive loss and metabolic disorders. Neurodegenerative disorders, such as Alzheimer’s disease (AD), and metabolic disease, such as diabetes mellitus (DM), are intimately linked with underlying cellular mechanisms that can determine the course of these disorders and include the inverse relationship with autophagy and the mechanistic target of rapamycin (mTOR) that can involve transient receptor potential cation channel subfamily V member (1/2) (TRPV). Autophagy, mTOR, and TRPV can oversee a number of cellular functions that involve mitochondrial integrity, apolipoprotein E-*ε*4 (APOE-*ε*4), inflammation, and oxidative stress. Oversight of these pathways by CBD and GLP-1 receptor agonists can be translated into improved weight management, glucose homeostasis, cognitive health, and improved liver function. The figure was completed using the Microsoft PowerPoint software (version 16.103.1).

CBD can oversee pathways of oxidative stress, inflammation, and programmed cell death mechanisms of autophagy [21–24] (Fig. 1). In AD models, CBD can lead to tau degradation though activation of autophagy pathways that may assist in the early stages of AD treatment [22]. CBD also may foster microglial phagocytosis of A*β* though the transient receptor potential cation channel subfamily V member 2 (TRPV2) channel activation and autophagy induction [25]. Of note, the TRPV1 family receptors are known to also affect oxidative stress, cellular survival, cellular metabolism [26–28]. In regard to metabolic disease, CBD derivatives can limit inflammation and offer cytoprotection in the liver, pancreas, and adipose tissue in experimental models of pre-diabetes and nonalcoholic fatty liver disease (NAFLD) [29], also known as metabolic dysfunction-associated steatotic liver disease (MASLD). In addition, CBD can reduce inflammatory macrophages and resolved metabolic dysfunction [30] as well as DM neuropathy [31]. These beneficial effects of CBD during metabolic disease can involve improved function of mitochondria and control of programmed cell death pathways of mitophagy [32] and autophagy [31].

As an underlying cellular pathway for both cognitive neurodegenerative disorders and metabolic disease, autophagy oversight by CBD may be critical for the benefits of this agent in AD and DM (Fig. 1). Under some conditions, loss of the autophagy pathway can cause memory loss in AD with the DM progression [33]. In contrast, during activation of autophagy, oxidative stress can be reduced, tau and A*β* deposition can be limited, myelin repair can be fostered, and memory function can be improved [34–37]. With metabolic disease, autophagy activation can be necessary for fatty acid metabolism during obesity [38], required to reduce cerebral ischemia during DM [39], and necessary to assist with tissue regeneration of muscle [40]. Yet, not all conditions of autophagy activation may offer cellular protection. Depending on the degree of cellular autophagy activation, unwanted effects may ensue such as mitochondrial dysfunction [41] and a decrease in neuronal repair mechanisms [42]. In addition, with different periods of autophagy flux, diabetic retinopathy may progress under these scenarios [43].

With the pathways of CBD and autophagy in mind for neurodegeneration and metabolic disease, one is inspired to consider how these compare to the cellular pathways of recently improved FDA treatments for the treatment of glucose homeostasis and obesity in DM that can advocate future clinical utility for CBD. In this regard, one can consider the glucagon-like peptide-1 (GLP-1) receptor agonists, such as semaglutide and tirzepatide, and the potential applications of these agents for dementia and neurodegenerative disorders [44]. Recently, semaglutide also was FDA approved for the treatment of metabolic disorders involving NAFLD or MASLD [45,46]. GLP-1 receptors are G protein-coupled receptors that are transmembrane proteins consisting of 7 *α*-helical transmembrane domains, an intracellular C-terminus, and an extracellular N-terminus. GLP-1 receptors exist on brain neurons and on *β*-cells of the pancreas, providing support for these receptors to affect both neurodegenerative and metabolic processes [47]. In addition, recent work suggests that GLP-1 agonists can have a high affinity binding for CBD receptors in the endocannabinoid system indicating a cross-reactivity [48]. Corresponding to CBD cellular pathways, agonism of GLP-1 receptors are also is involved in the control of the programmed cell death pathways of autophagy [49,50]. For example, exendrin-4, a GLP-1 agonist, can reduce hypoglycemic effects in the liver [51] and offer neuroprotection in the nervous system through the activation of autophagy to prevent diabetic retinopathy [52]. Yet, as previously mentioned, the induction of autophagy may require a fine modulation of autophagy flux levels. At times, reduced activation of autophagy may be necessary to offer cellular protection and this can occur through the mechanistic target of rapamycin (mTOR) [34,53]. mTOR is a 289-kDa serine/threonine protein kinase that is critical for the modulation of neurodegenerative and metabolic pathways and acts inversely with autophagy. In cases when a reduced activity of autophagy is required, activation of mTOR appears necessary. In experimental models of obesity, agonism of GLP-1 receptors can improve cognitive deficits and depression with the activation of mTOR and the inhibition of autophagy [54]. In addition, GLP-1 and TRPV1 activation that can impact mTOR have been shown to improve glucose homeostasis [55]. Similarly, CBD can employ the activation of mTOR with reduction in autophagy activity in experimental models of multiple sclerosis for neuroprotection and antiviral infection control [7,21,24]. Furthermore, CBD may utilize mTOR for cellular protection and limit oxidative stress through the TRPV1/2 family [26–28].

## Summary and Commentary

Neurodegenerative disease, especially cognitive loss such as AD, and metabolic disorders that include DM impact a significant portion of the global population, are growing in prevalence with increases in lifespan, and have significant financial consequences for global economies. Interestingly, there exists a close interplay for the underlying cause that foster neurodegenerative disease and metabolic disorders, such that CBD may represent an innovative therapy to treat cognitive loss that is tied to metabolic disease (Fig. 1). Neurodegenerative disorders that involve cognition and AD are becoming increasingly recognized for their dependence on metabolic disease, such as with DM, APOE-*ε*4, lipid, and cholesterol management. CBD can oversee cellular pathways involving oxidative stress, inflammation, and mitochondrial function. These pathways are ultimately linked to the programmed cell death pathway of autophagy, mTOR, and TRPV that are also shared with the pathways of recently FDA approved GLP-1 receptor agonists that provide treatment for DM, weight management, liver disease, and potential future applications for cognitive loss with AD. The mechanistic treatment similarities between CBD and GLP-1 receptor agonists as well as the ability of GLP-1 agonists possessing high affinity binding for CBD receptors provides exciting support for the future clinical applications of CBD in the nervous and metabolic systems. Yet, given the fine cellular oversight needed for the biological flux of the autophagy and mTOR pathways, future focused clinical translational studies are warranted to successfully develop effective treatments with CBD as well as for GLP-1 agonists for both cognitive loss and metabolic disease.
